# LC-MS and GC-MS based metabolomics platform for cancer research

**DOI:** 10.1186/2049-3002-2-S1-P41

**Published:** 2014-05-28

**Authors:** Wenyun Lu, Sisi Zhang, Xin Teng, Eugene Melamud, Mitchell A Lazar, Eileen White, Joshua D Rabinowitz

**Affiliations:** 1 Lewis-Sigler Institute for Integrative Genomics and Department of Chemistry, Princeton University, Princeton, NJ 08544, USA; 2 Rutgers Cancer Institute of New Jersey, New Brunswick, NJ 08903, USA; 3 Diabetes Research Center, University of Pennsylvania, Philadelphia, PA 19104, USA

## 

We present here a LC-MS and GC-MS based analytical platform for the comprehensive analysis of cellular metabolites, including water-soluble metabolites, and water-insoluble fatty acids, and phospholipids. The entire workflow consists of metabolic extraction, LC-MS and GC-MS runs, data analysis and interpretation. Standard operation procedures have been developed for the metabolite extraction from cell culture, tissue and serum/plasma, which involve liquid extraction using appropriate extraction solvents. Metabolites were analyzed using multiple analytical methods on multiple dedicated instruments. Cationic water-soluble metabolites were analyzed on a triple quadrupole instrument using hydrophilic interaction chromatography [1]. Anionic water-soluble metabolites were analyzed using high resolution ‘Exactive’ Orbitrap mass spectrometer coupled to reversed phase ion pairing chromatography [2]. Fatty acids and phospholipids were analyzed using Agilent Q-TOF instrument coupled to reversed phase chromatography [3]. Data analyses were performed using MAVEN program which converts the raw data into a validated table of metabolite-specific signals [4]. Examples will be provided to demonstrate our capability to analyze a broad range of metabolites from real biological samples, as well as to probe the metabolic fluxes using stable isotope tracers.
